# Thyroid Hormones and Metabolism Regulation: Which Role on Brown Adipose Tissue and Browning Process?

**DOI:** 10.3390/biom15030361

**Published:** 2025-03-02

**Authors:** Laura Sabatino, Cristina Vassalle

**Affiliations:** 1 Institute of Clinical Physiology, National Council of Research, 56124 Pisa, Italy; 2 Fondazione G. Monasterio, Regione Toscana, 56124 Pisa, Italy; cristina.vassalle@ftgm.it

**Keywords:** thyroid hormones, metabolism, brown adipose tissue, browning

## Abstract

Thyroid hormones (THs) are important modulators of many metabolic processes, being strictly associated with the control of energy balance, mainly through activities on the brain, white and brown adipose tissue, skeletal muscle, liver, and pancreas. In this review, the principal mechanisms of TH regulation on metabolic processes will be discussed and THs’ relevance in metabolic disease progression will be evaluated, especially in the cardiovascular context and correlated diseases. Moreover, we will discuss THs’ regulatory role on metabolic events in white and brown adipose tissue, with a special focus on the process of “browning”, which consists of the gradual acquisition by white adipocytes of the physical and functional characteristics of brown adipocytes. The advancements in research on molecular mechanisms and proposed physiopathological relevance of this process will be discussed.

## 1. Introduction

Thyroid hormones (THs) regulate metabolic processes important for normal growth and development and play essential regulatory roles in metabolism [1]. It is well known that TH status is strictly associated with energy expenditure [2]. Under physiological conditions, the intact hypothalamic–pituitary–thyroid (HPT) axis keeps THs’ circulating levels and energy homeostasis stable. The high levels of THs in hyperthyroidism prompt a hypermetabolic state, characterized by increased resting energy expenditure, weight loss, reduced cholesterol levels, increased lipolysis, and gluconeogenesis [3,4]. Conversely, in hypothyroidism, the reduction of TH levels promotes hypometabolic condition, characterized by reduced resting energy expenditure, weight gain, increased cholesterol levels, reduced lipolysis, and reduced gluconeogenesis [5,6]. This review will evaluate TH mechanisms mediating metabolic regulation and will consider how TH disturbances may affect lipid and glucose metabolism, blood pressure, and body weight, all aspects strongly associated with various metabolic parameters and whose alteration may induce the development of new diseased conditions or worsening of preexisting maladaptive events associated with metabolic dysfunctions. Furthermore, we will provide an overview of adipose tissue metabolism in physiological and disease conditions and discuss the regulatory role of THs in metabolic processes involving white and brown adipocytes. A particular focus will be given to the process of browning in mammalian white adipocytes, highlighting the latest research progress and the hypotheses of therapeutic relevance in humans.

## 2. General Aspects of TH Metabolism

**Figure 1:**
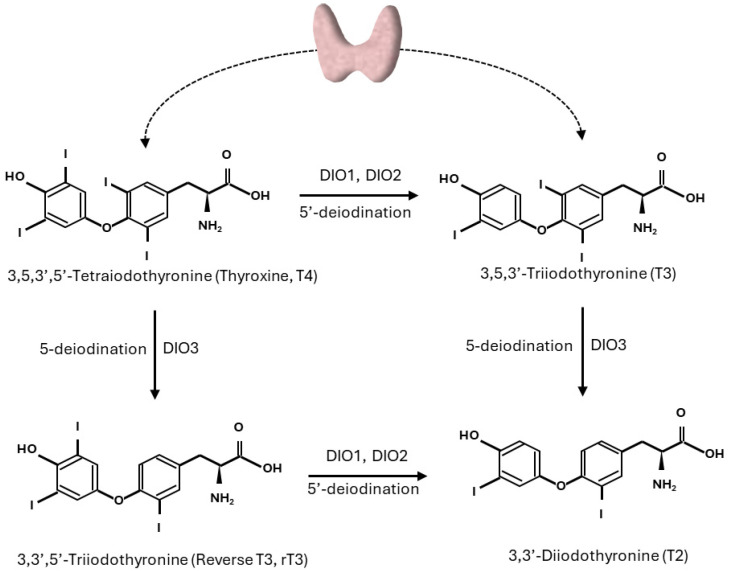
Pathways of outer and inner ring deiodination of thyroid hormones.

THs are key hormones in the regulation of metabolism in mammals, and recent advances in metabolomics and genomics have allowed a deeper understanding of THs’ global impact on mammalian physiology in peripheral tissues. THs are released into circulation by the thyrocytes of the thyroid gland as the final part of the HPT axis. Circulating TH concentrations, in turn, can inhibit both the thyrotropin-releasing hormone (TRH) produced by the hypothalamus and thyroid-stimulating hormone (TSH) secreted by the pituitary gland via negative feedback regulation [7]. Both thyroxine (T4) and triiodothyronine (T3) produced by the thyroid are transported in blood by carrier proteins that serve as TH reservoirs [8]. THs bound to these proteins are inactive and in a dynamic equilibrium with the circulating levels of free T4 (FT4) and free T3 (FT3), which instead enter the cells of the target tissues, exploiting the THs’ biological effects. The entrance of free THs into the target cells is mediated by several membrane transporters, such as monocarboxylate transporter 8 or 10 (MCT8 or MCT10), the organic anion-transporting polypeptide (OATP)1C1, and L-type amino acid transporters (LAT1 and LAT2) [9,10,11]. T4 is generally considered a pre-hormone, whereas T3, obtained by the deiodination of T4, is the biologically active TH. The enzymes regulating the activation/deactivation of these THs are the deiodinases (DIOs), which are selenoproteins involved in THs’ fine-tuning of the intracellular concentration and the biological activity of THs. Of the three known DIOs, DIO1 and DIO2 convert T4 to active T3, whereas DIO3 inactivates T3 to 3,3′-diiodo-L-thyronine (3,3′-T2) and T4 to inactive reverse triiodothyronine (rT3) [12] (Figure 1).

Once inside the cells, THs diffuse to the nucleus and bind to the thyroid hormone receptors (TRs) TRα and TRβ. Genes encoding the TRs, TRα and TRβ, and the TR proteins show a variety of expression within the target tissues, suggesting a specific tissue-dependent role for each TR isoform; interestingly, T3 binds TRs with a 10-fold higher affinity with respect to T4 [13]. Upon binding to T3, TR heterodimerizes with another nuclear hormone receptor (retinoic acid X receptor, RXR) and the newly formed complex is ready to interact with regulatory sequences in the promoters of target genes (TH response elements, TREs); specific cofactors are then recruited and the regulation of gene expression proceeds [14,15].

TH actions requiring interaction at the nuclear level are called “genomic”, whereas extranuclear biological actions of THs, not requiring a direct interaction with nuclear elements, are called “non-genomic” and involve binding with cell membrane receptors (such as integrins) and binding with cytosolic proteins. Furthermore, THs other than T3 and their metabolites, such as T4, rT3, 3,5-T2, etc., may also be involved in non-genomic actions [16].

## 3. TH Effects on Cardiovascular Metabolism and Correlated Diseases

THs control metabolism and prominently affect cardiovascular pathophysiology [17]. To note, the level of TSH can vary within the same subjects due to several determinants (e.g., age, circadian rhythm, genetic characteristics, and iodine intake) [3]. Thus, although reference ranges are based on fixed percentiles of the population distribution, it remains difficult to define whether a certain TSH value measured in an individual at a given time should be classified as normal at the population level. In fact, a high TSH concentration, even within the “euthyroid” reference range, is related to cardiovascular risk factors (e.g., BMI, lipid profile, hypertension, and metabolic syndrome) [18]. TSH is a useful screening test to assess the functioning of the HPT axis; however, the ratio between the hormones released by the thyroid gland (T3/T4) or FT3/FT4, the free fractions present in the bloodstream, has been introduced as a more reliable tool to evaluate the tissue-specific deiodinase activity in the conversion of T4 to T3 [19].

### 3.1. Obesity

It is well known that the thyroid has a role in the control of basal metabolic rate, energy expenditure, and thermogenesis and in the metabolism of carbohydrates, lipids, and proteins [4]. Nonetheless, the precise causes underlying the association between thyroid and obesity, as well as the reasons for the increase of TSH in obesity, are not clearly understood, but surely represent the result of a complex interaction of different abnormalities (Table 1).

In view of the well-known complex relationship between the thyroid, obesity, and metabolism, a correlation between thyroid and obesity has been largely evidenced in clinical studies; generally, hypothyroidism (underactive thyroid) induces a decrease of the body’s metabolism, resulting in weight gain, whereas hyperthyroidism (overactive thyroid) may cause weight loss [24,25]. Accordingly, a recent meta-analysis (total participants *n* = 107,734 for cross-sectional studies, *n* = 22,010 for longitudinal studies, *n* = 80 for RCTs) evidenced that an increasing TSH concentration was associated with weight gain, whereas increasing FT4 values were associated with weight loss [26].

Moreover, changes in TSH, FT3, FT4, and FT3/FT4 ratio values were associated with anthropometric measures changes, representing determinants of BMI and waist circumference variation [26,27,28]. Interestingly, from a clinical application point of view, interventional studies have suggested that these associations may be reversible; thus, some thyroid drugs may be active in terms of BMI changes [29,30,31].

### 3.2. Type 2 Diabetes (T2D)

T2D can be worsened by thyroid dysfunction, as well as reciprocally T2D can drive thyroid abnormalities, with the development of insulin resistance representing a key factor in the relationship between T2D and thyroid disorders [32].

Alterations in glucose metabolism are closely linked to changes in thyroid parameters, and indeed T2D often occurs with thyroid dysfunction (both hypothyroidism and hyperthyroidism); accordingly, it has been estimated that the prevalence of thyroid alterations is very frequent in T2D patients, especially hypothyroidism, and the risk increases with aging, primarily in females [33,34,35]. T2D may induce a TSH reduction, thus contrasting the conversion of T4 to T3 at peripheral levels. Moreover, insulin resistance and hyperinsulinemia promote thyroid tissue proliferation, nodule formation, and goiter size. Conversely, both hyper- and hypothyroidism have been associated with insulin resistance, being one key factor causing impaired glucose homeostasis in clinical settings [17,36]. The thyroid can worsen glycemic control in T2D (with the development of hypoglycemia in hypothyroidism and ketoacidosis in thyrotoxicosis), whereas reciprocally, poor glycemic control may have a role in the development of thyroid dysfunction in T2D patients [37,38]. In view of this reciprocal association, some antidiabetic drugs may influence thyroid function (e.g., metformin can reduce TSH) and TH analogues can affect glycemic concentration (KB141 and MB07811 reduced glycemia in mice fed a high-fat diet for 2 weeks) [39]. Many different mechanisms can explain the reciprocal pathophysiological relationship between the thyroid and T2D, and the main mechanisms in the relationship between the thyroid and T2D are reported in Table 2.

### 3.3. Dyslipidemia

TH may affect lipid metabolism by acting on different mechanisms [17,49]. Hypothyroidism has been associated with an adverse lipid profile (e.g., high total cholesterol, TC; triglycerides, TGs; lipoprotein(a), Lp(a); and low-density lipoprotein, LDL) and dysfunctional high-density lipoprotein (HDL) particles [50]. Conversely, hyperthyroidism is characterized by decreases in TC, LDL cholesterol, and Lp(a) levels [51]. However, the relationship between THs and HDL is even more complicated, HDL being increased, normal, or decreased in both hypothyroidism and hyperthyroidism [52,53].

Interestingly, some interventional studies employing thyroid treatment have beneficial effects on the lipid profile. Accordingly, mean serum TC and LDL cholesterol concentrations were observed to decrease following T4 therapy in subjects with mild thyroid dysfunction, although there were no significant effects on HDL or TG levels [54]. Moreover, therapy with levothyroxine in hypothyroid patients may improve the lipid profile (TC, LDL, and TGs), thus reducing the associated cardiovascular risk [55]. The main mechanisms in the relationship between the thyroid and dyslipidemia are reported in Table 3.

### 3.4. THs and NAFLD/NASH

THs regulate many metabolic activities in the liver, promoting export and oxidation of lipids and lipogenesis. Furthermore, THs control hepatic insulin sensitivity and suppress hepatic gluconeogenesis.

Since the HPT axis plays a fundamental role in metabolic pathways involving lipids and carbohydrates, the existence of a link between TH dysfunction and liver diseases has been explored over the years; in particular, the relationship between non-alcoholic fatty liver disease (NAFLD), also known as metabolic dysfunction-associated steatotic liver disease (MASLD), and hypothyroidism has recently attracted growing interest in the scientific community.

A range of progressive liver diseases are included in NAFLD, ranging from simple steatosis to NASH (non-alcoholic steatohepatitis). NASH is marked by >5% hepatic steatosis, with inflammation, hepatocyte destruction, and fibrosis which may potentially progress to advanced liver disease, cirrhosis, and hepatocellular carcinoma. Nowadays, NASH, together with chronic hepatitis C, is considered a major indicator for liver transplantation. Both genetic and environmental risk factors may contribute to NAFLD/NASH affirmation and worsening, such as T2DM, dyslipidemia, hypertension, metabolic syndrome, and thyroid disorders [60,61].

Several studies and meta-analyses have been conducted to define the possible association between NAFLD/NASH and thyroid function parameters/hypothyroidism and interesting conclusions have been drawn, underlining the positive association of hypothyroidism with the risk of NAFLD and the relevance of increased TSH concentration levels as a risk factor determining an increased incidence of NAFLD [62,63].

Based on THs’ effects on lipid metabolism and hepatic steatosis, the use of exogenous THs, in the form of LT4, regularly adopted to treat hypothyroidism, was evaluated in some studies in order to reduce the prevalence of NAFLD in the population studied [64,65]. Despite some benefits observed in patients, TH administration cannot be considered the resolutive therapeutic approach, given their systemic potentially harmful effects.

Additionally, TH analogs that specifically activate TRβ have been developed. TRβ is the most abundant TR in hepatocytes [66] and is known to be responsible for regulating those metabolic pathways in the liver that are frequently impaired in NAFLD and NASH [67]. Recently, The New England Journal of Medicine reported the results from a phase 3 multinational, double-blind, randomized, placebo-controlled trial on the employment of Resmetirom (MGL-3196), a selective liver TRβ agonist, developed for treatment of NASH, assessing the efficacy and safety in adults with confirmed diagnosis of NASH [68]. It is noteworthy that Resmetirom does not affect thyroid function and is well tolerated, which, at the moment, makes it the best option for therapeutical treatment of NASH. In fact, by accelerated approval pathway, in 2024 FDA approved Rezdiffra (Resmetirom) as the first (and only) approved treatment for NASH with moderate to advanced liver scarring (fibrosis), without cirrhosis of the liver, along with diet and exercise.

### 3.5. Hypertension

Overt and subclinical hyper- and hypothyroidism can all induce hypertension. Hyperthyroidism induces metabolic and hemodynamic alterations; endothelial dysfunction occurs and heart rate, pulse amplitude, cardiac output, and arterial stiffness increase, as well as erythropoietin production (with increase in red blood cell mass, blood volume, and cardiac preload), causing an increased cardiac output and hypertension [69].

Nonetheless, the available data have ineffectively demonstrated the association between subclinical hyperthyroidism and hypertension, which remains controversial at clinical levels [70,71].

In hypothyroidism, systemic vascular resistance increases and diastolic dysfunction occurs, as well as endothelial impairment (reduced nitric oxide bioavailability). Furthermore, dyslipidemia caused by compromised thyroid function is frequently found to aggravate vascular function [17].

Results from a meta-analysis in subjects with subclinical hypothyroidism (SCH; 10 studies/760 subjects related to flow-mediated dilatation of brachial artery and 23 studies/1521 subjects related to carotid intima-media thickness C-IMT) indicate that SCH is associated with endothelial dysfunction, whereas hypertension and dyslipidemia have a pivotal role (SCH associated with an increased diastolic blood pressure (DBP), systolic blood pressure (SBP), TGs, TC, and LDL cholesterol) [72].

Different studies have suggested that levothyroxine treatment may benefit blood pressure in SCH patients. Specifically, a meta-analysis (3 RCTs/117 patients) indicated that levothyroxine therapy in SCH patients can reduce C-IMT, following TC, TG, LDL, SBP, DBP, Lp(a) reduction, and flow-mediated dilatation improvement [73]. Another meta-analysis reported that levothyroxine treatment can improve blood pressure in patients with SCH [74]. Moreover, a further very recent meta-analysis (9 RCTs and 28 prospective cohorts) confirmed this observation [75]. The main effects of THs on blood pressure are reported in Table 4.

### 3.6. Metabolic Syndrome

The metabolic syndrome (MetS) definition includes different components, which, according to the National Cholesterol Education Program Adult Treatment Panel (NCEP-ATPIII) definition, consist of central obesity, hyperglycemia, hypertriglyceridemia, low HDL cholesterol, and hypertension. Thus, being the TH able to target the different components of MetS, the relationship between low–normal thyroid function and MetS has been reported in different epidemiological trials [80,81].

However, the relationship is complex (e.g., genetic factors may have a significant role and need further study, as recently observed) and still controversial (e.g., significant heterogeneity exists between available studies), as evidenced by recent meta-analyses, requiring further deepening in larger clinical trials as well as mechanistic studies in the future to better elucidate this relationship [82,83,84,85].

## 4. THs and Adipose Tissue Metabolism

### 4.1. General Characteristics of Adipose Tissue

Adipose tissue is a critical regulator of metabolic homeostasis, functioning as an energy depot and an endocrine organ capable of secreting biofactors (called adipokines) which regulate appetite, body fat distribution, insulin sensitivity and secretion, energy expenditure, and inflammation [86]. Adipose tissue is composed mainly by mature adipocytes and a stromal vascular fraction, which includes preadipocytes, fibroblasts, vascular smooth muscle, endothelial cells, resident monocytes and macrophages, lymphocytes, and adipose tissue-derived stem cells [87].

Adipose tissue consists of about 20–25% of total body weight in a healthy individual, and it can expand by increasing the volume (fat accumulation via lipogenesis) of pre-existing adipocytes (hypertrophy) and by generating new small adipocytes via proliferation and differentiation/adipogenesis (hyperplasia).

There are three types of adipose tissue, namely white (WAT), beige, and brown (BAT) adipose tissue. BAT is located in distinct anatomic regions in rodents (e.g., interscapular and supraclavicular regions) and humans (cervical, axillary, and paraspinal regions), while beige fat consists of brown-like adipocytes dispersed within classical white adipose tissue depots. Upon prolonged stimulation, such as cold exposure, BAT depots increase in both size and activity and the browning of WAT can occur, thus contributing to TG and fatty acid synthase catabolism and promoting energy expenditure. For this reason, the increase of BAT/beige adipocytes mass and/or activity could represent a strategy for promoting fat loss in obese populations [87].

WAT can be classified by location as either subcutaneous (located under the skin) or visceral/omental (located intra-abdominally, adjacent to internal organs). However, upon nutrient excess, fat accumulation can occur in ectopic areas, mainly within the visceral cavity, where it can influence the development of obesity-related comorbidities such as T2D and atherosclerosis [88]. WAT plays a key role in lipid storage, thus functioning as an energy reservoir for the other organs. During fasting and exercise, adipose tissue TGs are hydrolyzed to provide FAs for energy utilization by the rest of the body. The energy balance between storage and mobilization, which is controlled by nervous and endocrine stimuli, is crucial for whole-body homeostasis [89]. Metabolic stressors, such as high-fat feeding, cause dramatic changes in adipose tissue morphology, physiology, and cellular composition, thus leading to insulin resistance, dyslipidemia, and T2D [90].

### 4.2. TH Regulation of BAT Energy Expenditure

The adipose tissue is one of the main targets of THs, especially concerning the regulation of metabolic processes in energy expenditure. In homeothermic species, the processes regulating TH levels in the cells are very important in generating heat to maintain physiological body temperature [2].

**Figure 2:**
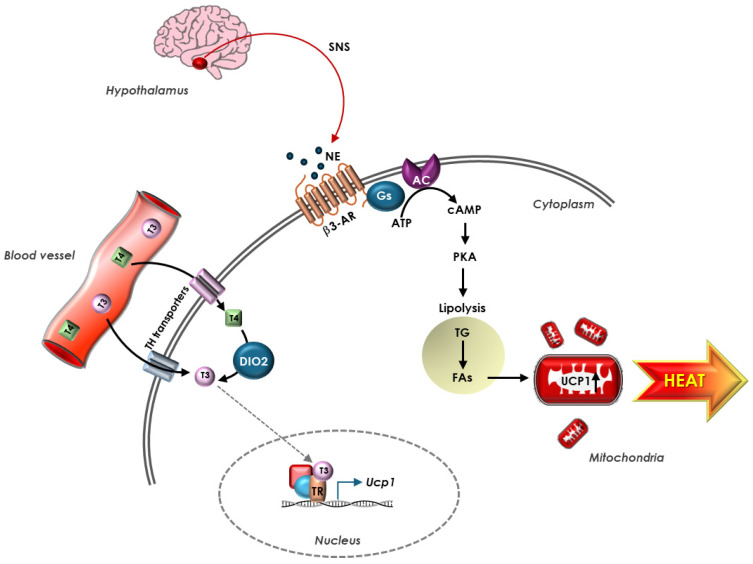
TH regulation of UCP1-dependent thermogenesis in BAT. Hypothalamus responds to stress signals rapidly releasing NE from terminal nerve of SNS present in BAT. NE stimulates β3-AR on adipocyte’s membrane, triggering a signaling cascade that leads to intracellular hydrolysis of TG, with consequent release of FAs. FAs, in turn, activate UCP1, which uncouples ATP production, and this causes release of energy as heat. Circulating THs enter the adipocytes through TH transporters in the cell membrane and T4 is converted into T3 by DIO2 enzyme. In the nucleus, upon binding TR, T3 stimulates UCP1 expression. In BAT, adrenergic and TH signaling coordinate to regulate UCP1 expression.

Exposure to cold stimulates the hypothalamus to activate processes leading to shivering, the first involuntary mechanism induced by adaptive thermogenesis in both adult humans and large mammals [91]. However, shivering mechanically provokes heat loss and is therefore considered a poor form of heat production, especially if compared to adaptive thermogenesis in the BAT of human newborns and other small mammals, where the increased metabolic rate does not involve shivering. Upon hypothalamus stimulation, adaptive thermogenesis requires the activation of the sympathetic nervous system and an augment of catecholamines throughout the body, particularly in the BAT, considered the main site of adaptive thermogenesis, especially in human newborns and smaller mammals, where the high surface-to-mass ratio makes the process more efficient [92]. BAT adipocytes contain vacuoles carrying TGs and are characterized by the presence of multiple mitochondria. Cells are surrounded by capillaries and are in contact with sympathetic nervous system (SNS) fibers, which transmit the nervous signal in response to the request of heat production. The norepinephrine released by the SNS interacts with adrenergic receptors (classically β3-AR but also β1- and β2-AR) in the brown adipocytes, triggering a signaling cascade that leads to intracellular hydrolysis of TGs, with the consequent release of FAs [93]. In mitochondria, FAs are oxidized and provide reduced substrates for the respiratory chain that pumps protons from the matrix to the inner membrane. In BAT, FAs activate uncoupling protein 1 (UCP1), which drives the re-entry into the matrix of protons not used for ATP synthesis, and this passage causes the release of energy as heat and the substrate oxidation, uncoupled by the synthesis of ATP [94]. T3, locally produced by the DIO2 enzyme, is one of the main stimulating agents of thermogenesis through the induction of UCP1; in the absence of T3, the thermogenic capacity of BAT is greatly reduced [95] (Figure 2).

Many studies on T3’s effects on UCP1 in brown adipocytes have been based on rodent models and, in cold-exposed rats, it was observed that T3 amplifies the adrenergic stimulation of UCP1 mRNA [96]. Several regions of the rat UCP1 promoter present TREs for TH–TR interaction and a relevant correlation has been observed between the occupancy of nuclear TRs and the increase in UCP1 expression [97]. Interesting information on TH–adrenergic system interactions in BAT thermogenesis was obtained by a mouse model with targeted disruption of the DIO2 gene. Despite normal T3 levels and increased T4 concentrations in plasma, these animals had functionally hypothyroid BAT, since the lack of DIO2-derived T3 did not permit an adequate thermogenic response. Moreover, in response to different adrenergic stimulants, brown adipocytes deprived of DIO2 exhibited a decreased cyclic adenosine monophosphate (cAMP) generation capacity due to reduced density of adrenergic receptors and the expression of adenylate cyclase, thus contributing to impaired thermogenesis [98]. Other research showed that, during cold exposure, the increased sympathetic stimulation of BAT provoked the activation of a compensatory mechanism in which relevant lipolysis and increase of cAMP-responsive genes led to impaired adaptive thermogenesis [99].

In humans, BAT is well recognized to be present in newborns as a defense mechanism to preserve adequate body temperature and, for the longest time, it has been debated whether BAT is rapidly lost within a few years after birth and almost totally disappears in adult life. However, in recent years, some findings have revealed the presence of active BAT also in adult humans. An initial understanding of human BAT was obtained from studies using combined positron emission tomography and computed tomography technologies, revealing the presence of significant deposits of active BAT, positive to ^18^F-fluorodeoxyglucose (^18^F-FDG) uptake, in a region extending from the anterior neck to the thorax and in which multilocular and immunopositive to UCP1 adipocytes were also detected [100]. More recently, this finding was confirmed by a non-invasive metabolic magnetic resonance imaging approach, which provided the advantage to perform longitudinal studies and to detect BAT also in thermoneutral conditions [101,102].

Since its identification in humans, BAT activation and thermogenesis have been investigated as potential targets for therapies oriented to the maintenance of body weight and amelioration of metabolic alterations, especially in obesity.

BAT thermogenic activity in humans is stimulated by cold exposure, and the sympathetic nervous system, which innervates BAT, is essential in this process. However, cold-induced BAT activation is impaired in obese people. The necessity of a safe and specific pharmacological approach for BAT activation brought initially to the development of sympathomimetic drugs, whose efficacy on weight loss was not as expected [103]. More recently, acute oral administration of mirabegron, a more selective β3-AR agonist, was shown to be associated with the metabolic activation of BAT in a dose-dependent way [104]; however, the increase in heart rate and blood pressure observed after its administration discouraged its use due to the risk of cardiovascular events in the long-term. Further compounds have been tested, even in association with mirabegron, such as pioglitazone, a PPARγ agonist [105], and chenodeoxycholic acid, a DIO2 activator through G protein–coupled bile acid receptor Gpbar1 (TGR5) [106].

### 4.3. WAT Browning: The Regulatory Role of TH

Besides WAT and BAT, well defined on the basis of their aspect and functions, there has been described a third population of adipocytes which have intermediate features between WAT and BAT and are therefore called beige adipose tissue. Beige adipocytes resemble brown adipocytes in morphology and function; in fact, they have multilocular lipid droplets, high mitochondrial content, and UCP1-expressing cells with thermogenic capacity. Therefore, beige adipocytes are considered brown-like cells that reside in WAT depots whose origin is still debated, since they might derive from the transformation of white adipocytes in response to adequate stimuli (such as cold or β3-adrenergic agonists), or from a subset of preadipocytes, or from tissue-resident progenitors [107]. It is currently accepted that beige adipocytes are important in weight control and, similarly to brown adipocytes, they affect the body’s energy balance. For these reasons, the mechanisms regulating the recruitment of beige adipocytes have received much interest in attempts to individuate a new therapeutic strategy for the treatment of obesity and T2D.

THs are considered among the principal browning agents, through both central and peripheral mechanisms [108]. Several studies on hyperthyroid rats demonstrated that THs play key regulatory activities on metabolism at central levels; in fact, when administered centrally, T3 inhibits AMP-activated protein kinase (AMPK) in the ventromedial nucleus of the hypothalamus (VMH), thus stimulating UCP1 expression through SNS mediation and leading to feeding-independent weight loss. This effect was abolished in UCP1-KO mice, confirming the role of UCP1 as the main regulator of TH effects at a central level [109,110]. Furthermore, in hyperthyroid rats, the genetic activation of AMPK totally blunted the effects of T3 on WAT browning, and the observed feeding-independent increase of body mass confirmed the thermogenic capacity of beige adipocytes in the reduction of body weight mediated by T3 administered at central level [111]. In addition to regulatory effects at central level, THs can also have direct effects on WAT, and data obtained from human multipotent adipose-derived stem cells after T3 treatment revealed the induction of TRβ1-dependent UCP1 expression [112]. Similarly, studies in mice showed that, after the administration of a TRβ1-specific agonist called GC-1, WAT browning was activated, eliciting an increase in metabolism, fat loss, and cold tolerance [113]. Furthermore, mice rendered hyperthyroid showed the presence of BAT markers in subcutaneous WAT [114] and, similarly, subcutaneous administration of T4 to rats induced mRNA expression of UCP1 and other BAT markers in gonads and subcutaneous WAT [111].

In humans, TH modulation of WAT browning was also investigated and a correlation between UCP1 mRNA expression and T4 levels was reported [111]. Moreover, data from human multipotent adipose-derived stem cells treated with T3 during differentiation showed an increase of UCP1 expression together with increased mitochondrial biogenesis [112]. Unfortunately, so far, only a limited number of studies are available on the effects of THs on browning in humans and no definitive conclusion can yet be drawn on the mechanisms involved.

An increased understanding of the morphological and functional characteristics of beige adipocytes, together with the regulatory mechanisms of browning, are of relevant interest, as the stimulation of the activity of this cell type in patients could potentially facilitate weight loss and improve metabolic health.

## 5. Conclusions

THs are important regulators of the principal metabolic processes in the body, providing a broad contribution to energy homeostasis in many relevant tissues. TH actions require the integration of metabolic pathways at both central and peripherical levels and, despite considerable advances in the understanding of the cellular and molecular mechanisms of TH regulation, much is still to be learned about their actions and targets.

Thyroid dysfunctions are closely interlinked with cardiovascular and metabolic diseases. Since abnormal TH levels negatively affect cardiovascular function, the rapid diagnosis and management of thyroid diseases are required in patients with cardiovascular risk to reduce associated complications and mortality. On the other side, great attention must be given to the thyroid system history of cardiovascular patients and an accurate choice of drugs used to treat cardiovascular diseases is necessary to avoid thyroid alteration or worsening. Moreover, THs are key hormones that regulate many metabolic processes which often require the coordination of multiple tissues under direct or indirect TH regulation.

Adipose tissue is a main target of thyroid hormones and constitutes the principal site for energy storage, acting as a regulator of energy balance and sending signals to maintain metabolic control. THs regulate many of the genetic markers involved in lipogenesis, lipolysis, and thermogenesis in BAT and, beyond that, THs can increase the thermogenic potential of BAT by stimulating the browning of WAT. In recent times, the study of the molecular signaling mechanisms involved in the browning process has become particularly challenging due to the growing interest in therapeutic drug development against obesity-related diseases.

However, further investigations are necessary to unravel the intricate biological features of various adipose tissue types and to elucidate the molecular pathways governing their interconversion.

## Figures and Tables

**Table 1:** Main mechanisms in the relationship between thyroid and obesity.

Mechanism	Consequences	References	Data Source
Development of obesity-related thyroid resistance in the peripheral tissues	Reduced expression of TSH receptors in adipose tissue of obese patients	[20,21]	In vitro humans
Increase of D1 gene expression and activity in subcutaneous and visceral adipose tissue of obese subjects	Increase of inflammatory cytokines (e.g., leptin), which stimulates the HPT axis Increase of TRH and TSH secretion	[22]	In vitro animals and humans Clinical studies
TH control on adipogenesis	Activation of PPARγ and C/EBPs	[23]	In vitro animals

TSH: thyroid stimulating hormone; HPT: hypothalamus-pituitary-thyroid; TRH: thyrotropin releasing hormone; PPARγ: peroxisome proliferator-activated receptor gamma; C/EBPs: CCAAT-enhancer-binding proteins.

**Table 2:** Main mechanisms in the relationship between the thyroid and T2D.

Mechanism	Consequences	References	Data Source
TH induces proinsulin gene expression by PI3K-AKT pathway	Glycogen synthesis Glucose uptake Gluconeogenesis	[40,41]	In vitro animals
T3 anti-apoptotic effects	Non-genomic activation of the AKT signaling pathway Maintenance of pancreatic islet structure, size, and consistency	[42]	In vitro humans
TH control of hepatic glucose metabolism	Increase GLUT2 expression	[43]	In vivo animals
T3 role in insulin sensitivity	Modulation of the GLUT4 gene Increase of basal and insulin-mediated glucose transport in skeletal muscle and adipocytes Increase of glucose oxidation rate and decrease of glycolytic intermediate F6P in cardiomyocytes Increase of mitochondrial biogenesis and pyruvate transport across the mitochondrial membrane.	[43,44]	In vivo and in vitro animals Clinical studies
T3 increases liver gluconeogenesis	Increase of PEPCK activity	[45]	In vivo animals
Genetic risk for T2D	Homozygosity for the Thr92Ala polymorphism of DIO2 gene	[46]	In vivo humans
TH directly controls insulin secretion by β-cells.	Reduced glucose-induced insulin secretion in hypothyroidism Increased β-cell response to glucose in hyperthyroidism	[47]	In vivo humans
T3 inverse relationship with ghrelin (gut hormone)	Effects on insulin sensitivity and islet cell proliferation	[48]	In vivo humans

PI3K: phosphoinositide 3-kinases; AKT: protein kinase B; GLUT2 and GLUT 4: glucose transporter type 2 and 4 gene; F6P: fructose 6-phosphate; PEPCK: phosphoenolpyruvate carboxykinase; DIO2: deiodinase type 2.

**Table 3:** Main mechanisms in the relationship between the thyroid and dyslipidemia.

Mechanism	Consequences	References	Data Source
T3 modulates the lipolysis/lipogenesis balance	Modulation of lipolytic enzymes (e.g., carnitine palmitoyltransferase 1α, ADPN, triacylglycerol lipase, and LPL) Modulation of lipogenic enzymes in liver and adipose tissue (e.g., acety-CoA-carboxylase, FA synthase, ME, and G6PDH)	[56]	In vivo animals
T3 regulates adipogenesis	C/EBPs and PPARγ	[57]	In vitro animals
TH and lipid-metabolizing enzymes	Effects on HMG-CoA reductase, HNF4A, 7α-hydroxylase, LPL, and PCSK9	[58]	In vivo and in vitro animals and humans
TH and lipoprotein receptors and lipid-associated transfer proteins	Effect on low-density lipoprotein receptor, ABCA1, CETP, and LCAT	[58,59]	In vivo and in vitro animals and humans

ADPN: adiponutrin; LPL: lipoprotein lipase; ME: malic enzyme; G6PDH: glucose-6-phosphate dehydrogenase; C/EBPs: CCAAT-enhancer-binding proteins; PPARγ: peroxisome proliferator-activated receptor gamma; HMG-CoA: 3-hydroxy-3-methyl-glutaryl-coenzyme A; HNF4A: hepatocyte nuclear factor-4a.

**Table 4:** Main mechanisms in the relationship between the thyroid and blood pressure.

Mechanism	Consequences	References	Data Source
TH effects on endothelial function	Ion channel activation (Na^+^, K^+^, Ca^2+^) Promotion of the production of nitric oxide Modulation of oxidative stress and inflammation reduction in systemic vascular resistance	[76,77,78]	In vitro, animals and humans
TH effects on heart	Modulation of gene expression (e.g., α-myosin heavy chain, β1-adrenergic receptor, and atrial natriuretic hormone) Chronotropic effect Cardiac output	[69]	In vivo humans
TH effects on kidney	Renal blood flow Glomerular filtration rate Electrolyte homeostasis Kidney structure	[79]	In vivo humans

## References

[1] Brent G.A. (2012). Mechanisms of thyroid hormone action. J. Clin. Investig..

[2] McAninch E.A., Bianco A.C. (2014). Thyroid hormone signaling in energy homeostasis and energy metabolism. Ann. N. Y Acad. Sci..

[3] Andersen S., Bruun N.H., Pedersen K.M., Laurberg P. (2003). Biologic variation is important for interpretation of thyroid function tests. Thyroid.

[4] Mullur R., Liu Y.Y., Brent G.A. (2014). Thyroid hormone regulation of metabolism. Physiol. Rev..

[5] Cheng S.Y., Leonard J.L., Davis P.J. (2010). Molecular aspects of thyroid hormone actions. Endocr. Rev..

[6] Iwen K.A., Schroder E., Brabant G. (2013). Thyroid hormones and the metabolic syndrome. Eur. Thyroid J..

[7] Magner J.A. (1990). Thyroid-stimulating hormone: Biosynthesis, cell biology, and bioactivity. Endocr. Rev..

[8] Janssen S.T., Janssen O.E. (2017). Directional thyroid hormone distribution via the blood stream to target sites. Mol. Cell Endocrinol..

[9] Groeneweg S., van Geest F.S., Peeters R.P., Heuer H., Visser W.E. (2020). Thyroid Hormone Transporters. Endocr. Rev..

[10] Friesema E.C., Ganguly S., Abdalla A., Manning Fox J.E., Halestrap A.P., Visser T.J. (2003). Identification of monocarboxylate transporter 8 as a specific thyroid hormone transporter. J. Biol. Chem..

[11] Friesema E.C., Jansen J., Jachtenberg J.W., Visser W.E., Kester M.H., Visser T.J. (2008). Effective cellular uptake and efflux of thyroid hormone by human monocarboxylate transporter 10. Mol. Endocrinol..

[12] Sabatino L., Vassalle C., Del Seppia C., Iervasi G. (2021). Deiodinases and the Three Types of Thyroid Hormone Deiodination Reactions. Endocrinol. Metab..

[13] Vella K.R., Hollenberg A.N. (2017). The actions of thyroid hormone signaling in the nucleus. Mol. Cell Endocrinol..

[14] Kurokawa R., Yu V.C., Naar A., Kyakumoto S., Han Z., Silverman S., Rosenfeld M.G., Glass C.K. (1993). Differential orientations of the DNA-binding domain and carboxy-terminal dimerization interface regulate binding site selection by nuclear receptor heterodimers. Genes. Dev..

[15] Paquette M.A., Atlas E., Wade M.G., Yauk C.L. (2014). Thyroid hormone response element half-site organization and its effect on thyroid hormone mediated transcription. PLoS ONE.

[16] Tedeschi L., Vassalle C., Iervasi G., Sabatino L. (2021). Main Factors Involved in Thyroid Hormone Action. Molecules.

[17] Pingitore A., Gaggini M., Mastorci F., Sabatino L., Cordiviola L., Vassalle C. (2024). Metabolic Syndrome, Thyroid Dysfunction, and Cardiovascular Risk: The Triptych of Evil. Int. J. Mol. Sci..

[18] Neves J.S., Fontes-Carvalho R., Borges-Canha M., Leite A.R., von Hafe M., Vale C., Martins S., Guimaraes J.T., Carvalho D., Leite-Moreira A. (2022). Association of thyroid function, within the euthyroid range, with cardiovascular risk: The EPIPorto study. Front. Endocrinol..

[19] Bianco A.C., da Conceicao R.R. (2018). The Deiodinase Trio and Thyroid Hormone Signaling. Methods Mol. Biol..

[20] Bell A., Gagnon A., Grunder L., Parikh S.J., Smith T.J., Sorisky A. (2000). Functional TSH receptor in human abdominal preadipocytes and orbital fibroblasts. Am. J. Physiol. Cell Physiol..

[21] Nannipieri M., Cecchetti F., Anselmino M., Camastra S., Niccolini P., Lamacchia M., Rossi M., Iervasi G., Ferrannini E. (2009). Expression of thyrotropin and thyroid hormone receptors in adipose tissue of patients with morbid obesity and/or type 2 diabetes: Effects of weight loss. Int. J. Obes..

[22] Walczak K., Sieminska L. (2021). Obesity and Thyroid Axis. Int. J. Environ. Res. Public. Health.

[23] Obregon M.J. (2008). Thyroid hormone and adipocyte differentiation. Thyroid.

[24] Taylor P.N., Medici M.M., Hubalewska-Dydejczyk A., Boelaert K. (2024). Hypothyroidism. Lancet.

[25] Lee S.Y., Pearce E.N. (2023). Hyperthyroidism: A Review. JAMA.

[26] Roa Duenas O.H., Xu Y., Ikram M.A., Peeters R.P., Visser E., Chaker L. (2024). Thyroid Function and Anthropometric Measures: A Systematic Review and Meta-Analysis. Endocr. Pract..

[27] Deng L., Zheng X., Shuai P., Yu X. (2023). Thyroid-Related Hormones Changes Predict Changes in Anthropometric Measures and Incidence of Obesity in Chinese Euthyroid Persons. Horm. Metab. Res..

[28] Nie X., Ma X., Xu Y., Shen Y., Wang Y., Bao Y. (2020). Characteristics of Serum Thyroid Hormones in Different Metabolic Phenotypes of Obesity. Front. Endocrinol..

[29] Gunasekaran K., Ng D.X., Tan N.C. (2024). Thyroid function status in patients with hypothyroidism on thyroxine replacement and associated factors: A retrospective cohort study in primary care. BMC Prim. Care.

[30] Sirigiri S., Vaikkakara S., Sachan A., Srinivasarao P.V., Epuri S., Anantarapu S., Mukka A., Chokkapu S.R., Venkatanarasu A., Poojari R. (2016). Correction of Hypothyroidism Leads to Change in Lean Body Mass without Altering Insulin Resistance. Eur. Thyroid J..

[31] Rathi M.S., Miles J.N., Jennings P.E. (2008). Weight gain during the treatment of thyrotoxicosis using conventional thyrostatic treatment. J. Endocrinol. Investig..

[32] Biondi B., Kahaly G.J., Robertson R.P. (2019). Thyroid Dysfunction and Diabetes Mellitus: Two Closely Associated Disorders. Endocr. Rev..

[33] Yadav A., Yadav G.A.M., Narsingrao K.K., Nanda Kumar L.G., Yadav G.S.N. (2021). Prevalence of thyroid disorders among patients with diabetes in rural South India. Diabetes Metab. Syndr..

[34] Jali M.V., Kambar S., Jali S.M., Pawar N., Nalawade P. (2017). Prevalence of thyroid dysfunction among type 2 diabetes mellitus patients. Diabetes Metab. Syndr..

[35] Vemula S.L., Aramadaka S., Mannam R., Sankara Narayanan R., Bansal A., Yanamaladoddi V.R., Sarvepalli S.S. (2023). The Impact of Hypothyroidism on Diabetes Mellitus and Its Complications: A Comprehensive Review. Cureus.

[36] Erion M.D., Cable E.E., Ito B.R., Jiang H., Fujitaki J.M., Finn P.D., Zhang B.H., Hou J., Boyer S.H., van Poelje P.D. (2007). Targeting thyroid hormone receptor-beta agonists to the liver reduces cholesterol and triglycerides and improves the therapeutic index. Proc. Natl. Acad. Sci. USA.

[37] Kalra S., Unnikrishnan A.G., Sahay R. (2014). The hypoglycemic side of hypothyroidism. Indian J. Endocrinol. Metab..

[38] Elgazar E.H., Esheba N.E., Shalaby S.A., Mohamed W.F. (2019). Thyroid dysfunction prevalence and relation to glycemic control in patients with type 2 diabetes mellitus. Diabetes Metab. Syndr..

[39] Cannarella R., Condorelli R.A., Barbagallo F., Aversa A., Calogero A.E., La Vignera S. (2021). TSH lowering effects of metformin: A possible mechanism of action. J. Endocrinol. Investig..

[40] Goulart-Silva F., Serrano-Nascimento C., Texeira S.S., Nunes M.T. (2013). Triiodothyronine (T3) induces proinsulin gene expression by activating PI3K: Possible roles for GSK-3beta and the transcriptional factor PDX-1. Exp. Clin. Endocrinol. Diabetes.

[41] Sano H., Kane S., Sano E., Miinea C.P., Asara J.M., Lane W.S., Garner C.W., Lienhard G.E. (2003). Insulin-stimulated phosphorylation of a Rab GTPase-activating protein regulates GLUT4 translocation. J. Biol. Chem..

[42] Verga Falzacappa C., Panacchia L., Bucci B., Stigliano A., Cavallo M.G., Brunetti E., Toscano V., Misiti S. (2006). 3,5,3′-triiodothyronine (T3) is a survival factor for pancreatic beta-cells undergoing apoptosis. J. Cell Physiol..

[43] Weinstein S.P., O’Boyle E., Fisher M., Haber R.S. (1994). Regulation of GLUT2 glucose transporter expression in liver by thyroid hormone: Evidence for hormonal regulation of the hepatic glucose transport system. Endocrinology.

[44] Mendez D.A., Ortiz R.M. (2021). Thyroid hormones and the potential for regulating glucose metabolism in cardiomyocytes during insulin resistance and T2DM. Physiol. Rep..

[45] Hanson R.W., Reshef L. (1997). Regulation of phosphoenolpyruvate carboxykinase (GTP) gene expression. Annu. Rev. Biochem..

[46] Dora J.M., Machado W.E., Rheinheimer J., Crispim D., Maia A.L. (2010). Association of the type 2 deiodinase Thr92Ala polymorphism with type 2 diabetes: Case-control study and meta-analysis. Eur. J. Endocrinol..

[47] Mohammed Hussein S.M., AbdElmageed R.M. (2021). The Relationship Between Type 2 Diabetes Mellitus and Related Thyroid Diseases. Cureus.

[48] Gjedde S., Vestergaard E.T., Gormsen L.C., Riis A.L., Rungby J., Moller N., Weeke J., Jorgensen J.O. (2008). Serum ghrelin levels are increased in hypothyroid patients and become normalized by L-thyroxine treatment. J. Clin. Endocrinol. Metab..

[49] Yetkin D.O., Dogantekin B. (2015). The Lipid Parameters and Lipoprotein(a) Excess in Hashimoto Thyroiditis. Int. J. Endocrinol..

[50] Su X., Peng H., Chen X., Wu X., Wang B. (2022). Hyperlipidemia and hypothyroidism. Clin. Chim. Acta.

[51] Feingold K.R., Feingold K.R., Anawalt B., Blackman M.R., Boyce A., Chrousos G., Corpas E., de Herder W.W., Dhatariya K., Dungan K., Hofland J. (2000). The Effect of Endocrine Disorders on Lipids and Lipoproteins.

[52] Janovsky C., Generoso G., Goulart A.C., Santos R.D., Blaha M.J., Jones S., Toth P.P., Lotufo P.A., Bittencourt M.S., Bensenor I.M. (2020). Differences in HDL particle size in the presence of subclinical thyroid dysfunctions: The ELSA-Brasil study. Atherosclerosis.

[53] Post A., Garcia E., Gruppen E.G., Kremer D., Connelly M.A., Bakker S.J.L., Dullaart R.P.F. (2022). Higher Free Triiodothyronine Is Associated With Higher HDL Particle Concentration and Smaller HDL Particle Size. J. Clin. Endocrinol. Metab..

[54] Danese M.D., Ladenson P.W., Meinert C.L., Powe N.R. (2000). Clinical review 115: Effect of thyroxine therapy on serum lipoproteins in patients with mild thyroid failure: A quantitative review of the literature. J. Clin. Endocrinol. Metab..

[55] Nishat S., Mueka I.N., Hassan M.U., Pandey R.K., Lwin B.B., Vashishta A., Nassar S.T. (2024). Effect of Levothyroxine Therapy on the Lipid Profile of Patients With Hypothyroidism: A Systematic Review. Cureus.

[56] Oppenheimer J.H., Schwartz H.L., Lane J.T., Thompson M.P. (1991). Functional relationship of thyroid hormone-induced lipogenesis, lipolysis, and thermogenesis in the rat. J. Clin. Investig..

[57] Obregon M.J. (2014). Adipose tissues and thyroid hormones. Front. Physiol..

[58] Wang X., Wu Z., Liu Y., Wu C., Jiang J., Hashimoto K., Zhou X. (2024). The role of thyroid-stimulating hormone in regulating lipid metabolism: Implications for body-brain communication. Neurobiol. Dis..

[59] Duntas L.H. (2002). Thyroid disease and lipids. Thyroid.

[60] Luukkonen P.K., Qadri S., Ahlholm N., Porthan K., Männistö V., Sammalkorpi H., Penttilä A.K., Hakkarainen A., Lehtimäki T.E., Gaggini M. (2022). Distinct contributions of metabolic dysfunction and genetic risk factors in the pathogenesis of non-alcoholic fatty liver disease. J. Hepatol..

[61] Younossi Z., Anstee Q.M., Marietti M., Hardy T., Henry L., Eslam M., George J., Bugianesi E. (2018). Global burden of NAFLD and NASH: Trends, predictions, risk factors and prevention. Nat. Rev. Gastroenterol. Hepatol..

[62] Guo Z., Li M., Han B., Qi X. (2018). Association of non-alcoholic fatty liver disease with thyroid function: A systematic review and meta-analysis. Dig. Liver Dis..

[63] Zeng X., Li B., Zou Y. (2021). The relationship between non-alcoholic fatty liver disease and hypothyroidism: A systematic review and meta-analysis. Medicine.

[64] Liu L., Yu Y., Zhao M., Zheng D., Zhang X., Guan Q., Xu C., Gao L., Zhao J., Zhang H. (2017). Benefits of Levothyroxine Replacement Therapy on Nonalcoholic Fatty Liver Disease in Subclinical Hypothyroidism Patients. Int. J. Endocrinol..

[65] Bruinstroop E., Dalan R., Cao Y., Bee Y.M., Chandran K., Cho L.W., Soh S.B., Teo E.K., Toh S.A., Leow M.K.S. (2018). Low-Dose Levothyroxine Reduces Intrahepatic Lipid Content in Patients with Type 2 Diabetes Mellitus and NAFLD. J. Clin. Endocrinol. Metab..

[66] Anyetei-Anum C.S., Roggero V.R., Allison L.A. (2018). Thyroid hormone receptor localization in target tissues. J. Endocrinol..

[67] Sinha R.A., Bruinstroop E., Singh B.K., Yen P.M. (2019). Nonalcoholic Fatty Liver Disease and Hypercholesterolemia: Roles of Thyroid Hormones, Metabolites, and Agonists. Thyroid.

[68] Harrison S.A., Bedossa P., Guy C.D., Schattenberg J.M., Loomba R., Taub R., Labriola D., Moussa S.E., Neff G.W., Rinella M.E. (2024). A Phase 3, Randomized, Controlled Trial of Resmetirom in NASH with Liver Fibrosis. N. Engl. J. Med..

[69] Berta E., Lengyel I., Halmi S., Zrinyi M., Erdei A., Harangi M., Pall D., Nagy E.V., Bodor M. (2019). Hypertension in Thyroid Disorders. Front. Endocrinol..

[70] Vidili G., Delitala A., Manetti R. (2021). Subclinical hyperthyroidism: The cardiovascular point of view. Eur. Rev. Med. Pharmacol. Sci..

[71] Volzke H., Ittermann T., Schmidt C.O., Dorr M., John U., Wallaschofski H., Stricker B.H., Felix S.B., Rettig R. (2009). Subclinical hyperthyroidism and blood pressure in a population-based prospective cohort study. Eur. J. Endocrinol..

[72] Gong N., Gao C., Chen X., Fang Y., Tian L. (2019). Endothelial Function in Patients with Subclinical Hypothyroidism: A Meta-Analysis. Horm. Metab. Res..

[73] Zhao T., Chen B., Zhou Y., Wang X., Zhang Y., Wang H., Shan Z. (2017). Effect of levothyroxine on the progression of carotid intima-media thickness in subclinical hypothyroidism patients: A meta-analysis. BMJ Open.

[74] He W., Li S., Zhang J.A., Zhang J., Mu K., Li X.M. (2018). Effect of Levothyroxine on Blood Pressure in Patients With Subclinical Hypothyroidism: A Systematic Review and Meta-Analysis. Front. Endocrinol..

[75] Darouei B., Amani-Beni R., Abhari A.P., Fakhrolmobasheri M., Shafie D., Heidarpour M. (2024). Systematic review and meta-analysis of levothyroxine effect on blood pressure in patients with subclinical hypothyroidism. Curr. Probl. Cardiol..

[76] Cooper D.S., Biondi B. (2012). Subclinical thyroid disease. Lancet.

[77] Kannan L., Shaw P.A., Morley M.P., Brandimarto J., Fang J.C., Sweitzer N.K., Cappola T.P., Cappola A.R. (2018). Thyroid Dysfunction in Heart Failure and Cardiovascular Outcomes. Circ. Heart Fail..

[78] Mancini A., Di Segni C., Raimondo S., Olivieri G., Silvestrini A., Meucci E., Curro D. (2016). Thyroid Hormones, Oxidative Stress, and Inflammation. Mediat. Inflamm..

[79] Agahi S., Amouzegar A., Honarvar M., Azizi F., Mehran L. (2024). Interrelationship between thyroid hormones and reduced renal function, a review article. Thyroid Res..

[80] Fan X., Yao Y., Chai S., Wang B., Xie Y., Jiang Y., Lin L., Li Y., Fan P., Luo W. (2024). Association between hypothyroidism and metabolic syndrome in Qinghai, China. Front. Endocrinol..

[81] Biondi B. (2023). Subclinical Hypothyroidism in Patients with Obesity and Metabolic Syndrome: A Narrative Review. Nutrients.

[82] Pleic N., Gunjaca I., Babic Leko M., Zemunik T. (2023). Thyroid Function and Metabolic Syndrome: A Two-Sample Bidirectional Mendelian Randomization Study. J. Clin. Endocrinol. Metab..

[83] Li T., Geng H., Wang Y., Wu Z., Yang S., Hu Y.Q. (2022). Causal Association of Thyroid Signaling with C-Reactive Protein: A Bidirectional Mendelian Randomization. Comput. Math. Methods Med..

[84] Alwan H., Ribero V.A., Efthimiou O., Del Giovane C., Rodondi N., Duntas L. (2024). A systematic review and meta-analysis investigating the relationship between metabolic syndrome and the incidence of thyroid diseases. Endocrine.

[85] Ding X., Zhao Y., Zhu C.Y., Wu L.P., Wang Y., Peng Z.Y., Deji C., Zhao F.Y., Shi B.Y. (2021). The association between subclinical hypothyroidism and metabolic syndrome: An update meta-analysis of observational studies. Endocr. J..

[86] Auger C., Kajimura S. (2023). Adipose Tissue Remodeling in Pathophysiology. Annu. Rev. Pathol..

[87] Chait A., den Hartigh L.J. (2020). Adipose Tissue Distribution, Inflammation and Its Metabolic Consequences, Including Diabetes and Cardiovascular Disease. Front. Cardiovasc. Med..

[88] Kwok K.H., Lam K.S., Xu A. (2016). Heterogeneity of white adipose tissue: Molecular basis and clinical implications. Exp. Mol. Med..

[89] Morigny P., Boucher J., Arner P., Langin D. (2021). Lipid and glucose metabolism in white adipocytes: Pathways, dysfunction and therapeutics. Nat. Rev. Endocrinol..

[90] Reddy P., Lent-Schochet D., Ramakrishnan N., McLaughlin M., Jialal I. (2019). Metabolic syndrome is an inflammatory disorder: A conspiracy between adipose tissue and phagocytes. Clin. Chim. Acta.

[91] Silva J.E. (2006). Thermogenic mechanisms and their hormonal regulation. Physiol. Rev..

[92] Yau W.W., Singh B.K., Lesmana R., Zhou J., Sinha R.A., Wong K.A., Wu Y., Bay B.H., Sugii S., Sun L. (2019). Thyroid hormone (T(3)) stimulates brown adipose tissue activation via mitochondrial biogenesis and MTOR-mediated mitophagy. Autophagy.

[93] Cannon B., Nedergaard J. (2004). Brown adipose tissue: Function and physiological significance. Physiol. Rev..

[94] Cioffi F., Gentile A., Silvestri E., Goglia F., Lombardi A. (2018). Effect of Iodothyronines on Thermogenesis: Focus on Brown Adipose Tissue. Front. Endocrinol..

[95] Bianco A.C., Maia A.L., da Silva W.S., Christoffolete M.A. (2005). Adaptive activation of thyroid hormone and energy expenditure. Biosci. Rep..

[96] Hernandez A., Obregon M.J. (2000). Triiodothyronine amplifies the adrenergic stimulation of uncoupling protein expression in rat brown adipocytes. Am. J. Physiol. Endocrinol. Metab..

[97] Bianco A.C., Silva J.E. (1987). Nuclear 3,5,3′-triiodothyronine (T3) in brown adipose tissue: Receptor occupancy and sources of T3 as determined by in vivo techniques. Endocrinology.

[98] de Jesus L.A., Carvalho S.D., Ribeiro M.O., Schneider M., Kim S.W., Harney J.W., Larsen P.R., Bianco A.C. (2001). The type 2 iodothyronine deiodinase is essential for adaptive thermogenesis in brown adipose tissue. J. Clin. Investig..

[99] Christoffolete M.A., Linardi C.C., de Jesus L., Ebina K.N., Carvalho S.D., Ribeiro M.O., Rabelo R., Curcio C., Martins L., Kimura E.T. (2004). Mice with targeted disruption of the Dio2 gene have cold-induced overexpression of the uncoupling protein 1 gene but fail to increase brown adipose tissue lipogenesis and adaptive thermogenesis. Diabetes.

[100] Cypess A.M., Lehman S., Williams G., Tal I., Rodman D., Goldfine A.B., Kuo F.C., Palmer E.L., Tseng Y.H., Doria A. (2009). Identification and importance of brown adipose tissue in adult humans. N. Engl. J. Med..

[101] Gifford A., Towse T.F., Walker R.C., Avison M.J., Welch E.B. (2016). Characterizing active and inactive brown adipose tissue in adult humans using PET-CT and MR imaging. Am. J. Physiol. Endocrinol. Metab..

[102] Cai Z., Zhong Q., Feng Y., Wang Q., Zhang Z., Wei C., Yin Z., Liang C., Liew C.W., Kazak L. (2024). Non-invasive mapping of brown adipose tissue activity with magnetic resonance imaging. Nat. Metab..

[103] Carey A.L., Formosa M.F., Van Every B., Bertovic D., Eikelis N., Lambert G.W., Kalff V., Duffy S.J., Cherk M.H., Kingwell B.A. (2013). Ephedrine activates brown adipose tissue in lean but not obese humans. Diabetologia.

[104] Cypess A.M., Weiner L.S., Roberts-Toler C., Franquet Elía E., Kessler S.H., Kahn P.A., English J., Chatman K., Trauger S.A., Doria A. (2015). Activation of human brown adipose tissue by a β3-adrenergic receptor agonist. Cell Metab..

[105] Finlin B.S., Memetimin H., Zhu B., Confides A.L., Vekaria H.J., El Khouli R.H., Johnson Z.R., Westgate P.M., Chen J., Morris A.J. (2021). Pioglitazone does not synergize with mirabegron to increase beige fat or further improve glucose metabolism. JCI Insight.

[106] Watanabe M., Houten S.M., Mataki C., Christoffolete M.A., Kim B.W., Sato H., Messaddeq N., Harney J.W., Ezaki O., Kodama T. (2006). Bile acids induce energy expenditure by promoting intracellular thyroid hormone activation. Nature.

[107] Kaisanlahti A., Glumoff T. (2019). Browning of white fat: Agents and implications for beige adipose tissue to type 2 diabetes. J. Physiol. Biochem..

[108] Weiner J., Hankir M., Heiker J.T., Fenske W., Krause K. (2017). Thyroid hormones and browning of adipose tissue. Mol. Cell Endocrinol..

[109] Alvarez-Crespo M., Csikasz R.I., Martinez-Sanchez N., Dieguez C., Cannon B., Nedergaard J., Lopez M. (2016). Essential role of UCP1 modulating the central effects of thyroid hormones on energy balance. Mol. Metab..

[110] Lopez M., Varela L., Vazquez M.J., Rodriguez-Cuenca S., Gonzalez C.R., Velagapudi V.R., Morgan D.A., Schoenmakers E., Agassandian K., Lage R. (2010). Hypothalamic AMPK and fatty acid metabolism mediate thyroid regulation of energy balance. Nat. Med..

[111] Martinez-Sanchez N., Moreno-Navarrete J.M., Contreras C., Rial-Pensado E., Ferno J., Nogueiras R., Dieguez C., Fernandez-Real J.M., Lopez M. (2017). Thyroid hormones induce browning of white fat. J. Endocrinol..

[112] Lee J.Y., Takahashi N., Yasubuchi M., Kim Y.I., Hashizaki H., Kim M.J., Sakamoto T., Goto T., Kawada T. (2012). Triiodothyronine induces UCP-1 expression and mitochondrial biogenesis in human adipocytes. Am. J. Physiol. Cell Physiol..

[113] Lin J.Z., Martagon A.J., Cimini S.L., Gonzalez D.D., Tinkey D.W., Biter A., Baxter J.D., Webb P., Gustafsson J.A., Hartig S.M. (2015). Pharmacological Activation of Thyroid Hormone Receptors Elicits a Functional Conversion of White to Brown Fat. Cell Rep..

[114] Weiner J., Kranz M., Kloting N., Kunath A., Steinhoff K., Rijntjes E., Kohrle J., Zeisig V., Hankir M., Gebhardt C. (2016). Thyroid hormone status defines brown adipose tissue activity and browning of white adipose tissues in mice. Sci. Rep..

